# Acute Hypoxemic Respiratory Failure Caused by Nonspecific Interstitial Pneumonia in Mixed Connective Tissue Disease: A Case Report

**DOI:** 10.7759/cureus.99922

**Published:** 2025-12-23

**Authors:** Syed M Naqvi, Amin Ur Rehman Nadeem, Jason Liu, Tyler Paul, Sara R Oliveira

**Affiliations:** 1 Pulmonary Medicine, Chicago Medical School/Rosalind Franklin University of Medicine and Science, North Chicago, USA; 2 Critical Care Medicine, Captain James A. Lovell Federal Health Care Center, North Chicago, USA; 3 Internal Medicine, Chicago Medical School/Rosalind Franklin University of Medicine and Science, North Chicago, USA

**Keywords:** acute hypoxemic respiratory failure, connective tissue disease-associated interstitial lung disease, immunosuppressive therapy intensive care unit, mixed connective tissue disease, nonspecific interstitial lung disease (nsip), systemic autoimmune disease

## Abstract

Mixed connective tissue disease (MCTD) is an autoimmune overlap condition characterized by features of systemic lupus erythematosus, systemic sclerosis, and polymyositis, frequently involving pulmonary manifestations. Interstitial lung disease (ILD) is one of its most serious complications and may present acutely with hypoxemic respiratory failure. Among the ILD patterns associated with MCTD, nonspecific interstitial pneumonia (NSIP) predominates and is characterized by diffuse ground-glass opacities, reticulation, and potential responsiveness to early immunosuppressive therapy. We describe a 42-year-old male active-duty service member who presented with acute hypoxemic respiratory failure and was ultimately diagnosed with MCTD-associated NSIP following bronchoscopy and confirmatory serologies. He demonstrated marked clinical and radiographic improvement after receiving high-dose corticosteroids and mycophenolate mofetil. This case highlights the importance of considering autoimmune ILD in patients with diffuse pulmonary opacities and unexplained hypoxemia.

## Introduction

Mixed connective tissue disease (MCTD), an autoimmune overlap condition characterized by high-titer anti-U1 ribonucleoprotein (U1 RNP) antibodies and clinical features resembling systemic lupus erythematosus, systemic sclerosis, and polymyositis, often presents with significant pulmonary involvement [1]. Interstitial lung disease (ILD) occurs in nearly half of patients and represents the leading cause of morbidity and mortality in MCTD [2]. Among the ILD subtypes, nonspecific interstitial pneumonia (NSIP) is the most frequently encountered pattern and is associated with ground-glass opacities, reticulation, and variable fibrosis on imaging [3].

Early recognition of NSIP is essential because timely immunosuppressive therapy can reverse inflammation and prevent irreversible fibrosis. However, NSIP often mimics infectious pneumonia, leading to diagnostic delays, inappropriate antimicrobial therapy, and progression of the underlying autoimmune disease [4,5]. Diagnostic evaluation requires integration of serologic markers, high-resolution CT (HRCT), and bronchoalveolar lavage (BAL) to exclude infection and histopathologic confirmation when necessary [6].

This case highlights an acute NSIP presentation as the first manifestation of MCTD, emphasizing the importance of early autoimmune evaluation in patients with diffuse lung opacities and persistent hypoxemia despite appropriate antibiotic therapy.

## Case presentation

The patient is a 42-year-old man with a five-pack-year smoking history, pulmonary nodules, deep vein thrombosis (DVT), chronic anemia, an alcohol use disorder, and a possible diagnosis of rheumatoid arthritis (RA). He presented to the emergency department after experiencing a two- to three-day history of worsening cough, dyspnea, and generalized weakness. Additionally, he reported an unintentional weight loss of 20 pounds over the past three months.

On arrival, the patient was afebrile. Blood pressure was slightly lower than expected for a healthy adult, and his heart rate was markedly elevated, consistent with significant tachycardia. Oxygen saturation showed significant hypoxemia despite supplemental oxygen via nasal cannula (Table 1). Physical examination revealed tachypnea, tachycardia, and bilateral crackles on lung auscultation.

**Table 1:** Presentation vital signs

Vital sign	Patient value	Normal range
Temperature	Afebrile	36.5-37.5 °C (97.7-99.5 °F)
Blood pressure	110s/60s	~120/80 mmHg
Heart rate	130 beats per minute	60-100 beats per minute
Oxygen saturation	85 % on 2 L nasal cannula	≥94 % on room air

Initial laboratory evaluation demonstrated severe anemia, hyponatremia, and marked elevation of liver enzymes consistent with transaminitis (Table 2). The anemia showed a macrocytic pattern with significant variability in red blood cell morphology, suggesting contributions from nutritional deficiency, chronic disease, or alcohol-related marrow suppression. Hyponatremia was consistent with systemic inflammation, reduced oral intake, or possible endocrine dysfunction. Liver enzyme elevation demonstrated a pattern often seen with alcohol-associated injury, systemic inflammation, or muscle involvement related to connective tissue disease. The D-dimer was elevated, which can reflect active systemic inflammation, impaired clearance in the setting of liver dysfunction, or underlying thrombotic risk in a patient with a known history of DVT.

**Table 2:** Laboratory investigations: initial workup

Test	Patient value	Flag	Reference range
Hemoglobin	6.8 g/dL	Low	13.5-17.5 g/dL (male)
Hematocrit	21.5 %	Low	41-53 % (male)
Mean corpuscular volume	105.7 fL	High	80-100 fL
Red cell distribution width	22.0 %	High	11.5-14.5 %
White blood cell count	10.0 K/µL	Normal	4.0-11.0 K/µL
Platelets	284 K/µL	Normal	150-450 K/µL
Sodium	129 mEq/L	Low	135-145 mEq/L
Potassium	5.0 mEq/L	High	3.5-5.0 mEq/L
Chloride	103 mEq/L	Normal	98-107 mEq/L
Creatinine	0.44 mg/dL	Low	0.7-1.3 mg/dL
Estimated glomerular filtration rate	137 mL/min	Normal	>60 mL/min
Calcium	9.4 mg/dL	Normal	8.5-10.5 mg/dL
Albumin	3.4 g/dL	Low	3.5-5.0 g/dL
Aspartate aminotransferase	273 U/L	High	10-40 U/L
Alanine aminotransferase	83 U/L	High	7-56 U/L
Alkaline phosphatase	44 U/L	Normal	44-147 U/L
Bilirubin total	0.30 mg/dL	Normal	0.1-1.2 mg/dL
Magnesium	1.8 mg/dL	Normal	1.7-2.2 mg/dL
T3 free	2.00 pg/mL	Low	2.1-4.2 pg/mL
T4 free	1.20 ng/dL	Normal	0.8-2.0 ng/dL
Thyroid-stimulating hormone	2.476 µIU/mL	Normal	0.3-5.0 µIU/mL
D-dimer	1744 ng/mL	High	<500 ng/mL
Urinalysis protein	10 mg/dL	Abnormal	Negative
Urinalysis nitrite	Negative	Normal	Negative
Urinalysis leukocyte esterase	Negative	Normal	Negative
Respiratory viral PCR panel	Not detected	Normal	Negative
Bacterial PCR panel	Not detected	Normal	Negative

Thyroid function testing was performed to assess for hypothyroidism as a potential contributor to the hyponatremia. Urinalysis demonstrated the presence of protein, which, although mild, may reflect systemic inflammation, early glomerular involvement related to autoimmune disease, or transient functional proteinuria associated with acute illness. In the context of connective tissue disease, low-level proteinuria can also signal early immune-mediated renal involvement and therefore warrants continued outpatient monitoring.

**Figure 1:**
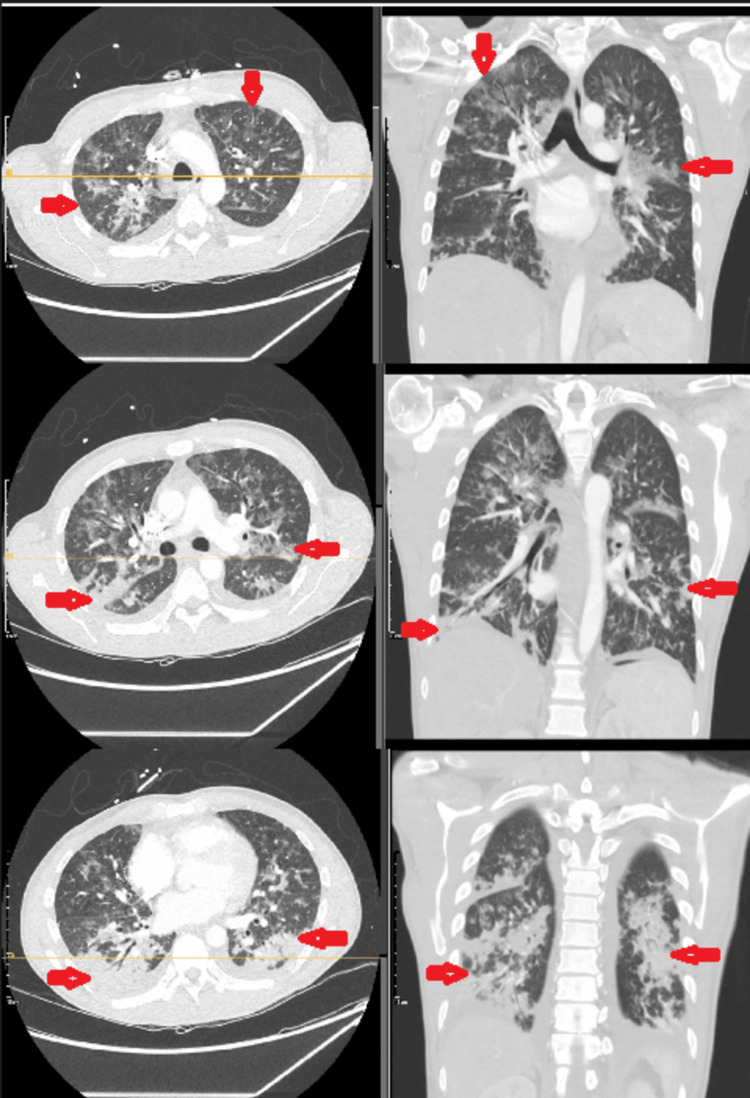
Axial and coronal views of chest CT angiography at three different levels, showing extensive bilateral consolidation, ground-glass, and nodular opacities (marked by red arrows)

A chest CT angiography scan ruled out pulmonary embolism but demonstrated extensive bilateral consolidation, ground-glass opacities, and nodular infiltrates, raising concern for ILD (Figure 1).

The patient received one unit of packed red blood cells and was empirically started on intravenous cefepime and doxycycline. He was initially admitted to the medical floor but was transferred to the intensive care unit the following morning due to increasing work of breathing, tachypnea, and worsening hypoxemia. Family history was notable for dermatomyositis in a first-degree relative, suggesting a genetic predisposition.

Bronchoscopy with transbronchial biopsies under fluoroscopic guidance was performed in the posterior segment of the right upper lobe, the lateral segment of the right middle lobe, and the lateral basal segment of the right lower lobe. Biopsy results revealed uniform interstitial inflammation and fibrosis consistent with NSIP. BAL was negative for bacterial, viral, and fungal pathogens, supporting a noninfectious inflammatory etiology. Laboratory evaluation showed elevated inflammatory markers, including procalcitonin, C-reactive protein, erythrocyte sedimentation rate, and ferritin levels (Table 3).

**Table 3:** Laboratory investigations: inflammatory markers

Test	Result	Flag	Reference range
Procalcitonin	0.350 ng/mL	Elevated	<0.05 ng/mL
C-reactive protein	42 mg/L	Elevated	<5 mg/L
Erythrocyte sedimentation rate	48 mm/hr	Elevated	<20 mm/hr (men)
Ferritin	1072.5 ng/mL	High	30-400 ng/mL

Rheumatology was consulted after an autoimmune serologic and rheumatologic workup, which returned positive for antinuclear antibody (ANA), anti-double-stranded DNA, anti-ribonucleoprotein (anti-RNP), anti-Smith/ribonucleoprotein (anti-Sm/RNP), anti-chromatin antibodies, and rheumatoid factor, with a negative cyclic citrullinated peptide IgG antibody (anti-CCP) (Table 4). This autoantibody profile, particularly the presence of anti-RNP and anti-Sm/RNP together with high-titer ANA, supports a possible diagnosis of MCTD, which is classically associated with antibodies targeting U1-RNP and overlapping features of several connective tissue diseases.

**Table 4:** Laboratory investigations: autoimmune serology and rheumatologic workup AI stands for Antibody Index. It is a standardized unit used by many autoimmune serology laboratories to report the quantitative level of an antibody: (1) AI < 0.9 → Negative, (2) AI 1.0-1.5 → Weak positive, and (3) AI > 1.5 → Strong positive. Different laboratories may have slightly different cutoffs, but the concept is the same. ANA, antinuclear antibody; anti-dsDNA, anti-double-stranded DNA; anti-RNP, anti-ribonucleoprotein; anti-Sm/RNP, anti-Smith/ribonucleoprotein

Test	Patient value	Flag	Reference range
Antinuclear antibodies screen	Positive	High	Negative
Anti-dsDNA antibody qualitative	Positive	High	Negative
Anti-dsDNA antibody quantitative	21 IU/mL	High	<10 IU/mL
Anti-RNP antibody qualitative	Positive	High	Negative
Anti-RNP antibody quantitative	3.4 AI	High	<0.9 AI
Anti-Sm/RNP antibody qualitative	Positive	High	Negative
Anti-Sm/RNP antibody quantitative	7.6 AI	High	<0.9 AI
Anti-chromatin antibody qualitative	Positive	High	Negative
Anti-chromatin antibody quantitative	1.7 AI	High	<0.9 AI
Anti-centromere B antibody qualitative	Negative	-	Negative
Anti-centromere B antibody quantitative	<0.2 AI	-	<0.9 AI
Anti-Jo1 antibody qualitative	Negative	-	Negative
Anti-Jo1 antibody quantitative	<0.2 AI	-	<0.9 AI
Anti-ribosomal P antibody qualitative	Negative	-	Negative
Anti-ribosomal P antibody quantitative	0.2 AI	-	<0.9 AI
Anti-Scl-70 antibody qualitative	Negative	-	Negative
Anti-Scl-70 antibody quantitative	<0.2 AI	-	<0.9 AI
Anti-Smith antibody qualitative	Negative	-	Negative
Anti-Smith antibody quantitative	0.4 AI	-	<0.9 AI
Anti-SSA/Ro antibody qualitative	Negative	-	Negative
Anti-SSA/Ro antibody quantitative	0.4 AI	-	<0.9 AI
Anti-SSB/La antibody qualitative	Negative	-	Negative
Anti-SSB/La antibody quantitative	<0.2 AI	-	<0.9 AI
Rheumatoid factor	182.1 IU/mL	High	<14 IU/mL
Cyclic citrullinated peptide IgG antibody	16 units	Normal	Negative <20 units; weak positive 20-39; moderate positive 40-59; strong positive ≥60
HIV 1/2 antibody/antigen screen	Nonreactive	Normal	Nonreactive
Complement component 3	74 mg/dL	Low	90-180 mg/dL
Complement component 4	16-27 mg/dL	Normal	10-40 mg/dL

In this context, the positive RF with a negative anti-CCP may reflect involvement of the MCTD spectrum rather than classic seropositive RA, because anti-CCP antibodies are much more specific for RA and are detected in only a minority of patients with MCTD. The patient had a similar serologic pattern of positive RF with negative anti-CCP several years earlier; at that time, additional autoimmune testing was not performed, and he declined medical management with methotrexate or hydroxychloroquine.

Given the clinical presentation, radiographic findings, biopsy results, and autoimmune serologies, and to address his acute symptoms, a treatment regimen was initiated beginning with intravenous methylprednisolone sodium succinate at a dosage of 60 mg every eight hours for two days. This was followed by a higher dosage of 500 mg daily for three days based on pulmonary recommendations. All cultures, including BAL samples tested for acid-fast bacilli, remained negative, supporting continued immunosuppressive treatment. The rheumatology team was also involved in the patient’s care and concurred with the management plan.

The patient’s respiratory status, including work of breathing and hypoxemia, continued to improve with corticosteroids, and he was weaned off supplemental oxygen. He was transitioned to oral prednisone at a dose of 60 mg daily with a tapering schedule. Mycophenolate mofetil (MMF) was initiated after confirming appropriate baseline studies, including a complete blood count, renal function panel, and hepatic function panel. MMF was started at 500 mg orally twice daily and gradually increased to a therapeutic dose of 1,000 mg twice daily for long-term management of suspected MCTD-associated NSIP. This treatment approach aligns with current recommendations from the American College of Rheumatology and the American College of Chest Physicians.

Transthoracic echocardiography was performed to rule out a cardiac cause of tachycardia. It revealed sinus tachycardia as fast as 120 beats per minute during the study, with normal overall and regional left ventricular systolic function, no valvular disease, no right heart disease, and no signs of pulmonary hypertension. Sinus tachycardia was considered secondary to anemia, anxiety, and acute illness and resolved as the patient improved.

He was discharged with close outpatient follow-up arranged with pulmonology, rheumatology, and gastroenterology for continued monitoring of his ILD and autoimmune disease and was scheduled for a colonoscopy as an outpatient to evaluate chronic anemia. Additionally, he reported bilateral lower extremity paresthesias suggestive of autoimmune-related neuropathy, for which gabapentin was initiated. Outpatient electromyography and neurology follow-up were recommended.

**Figure 2:**
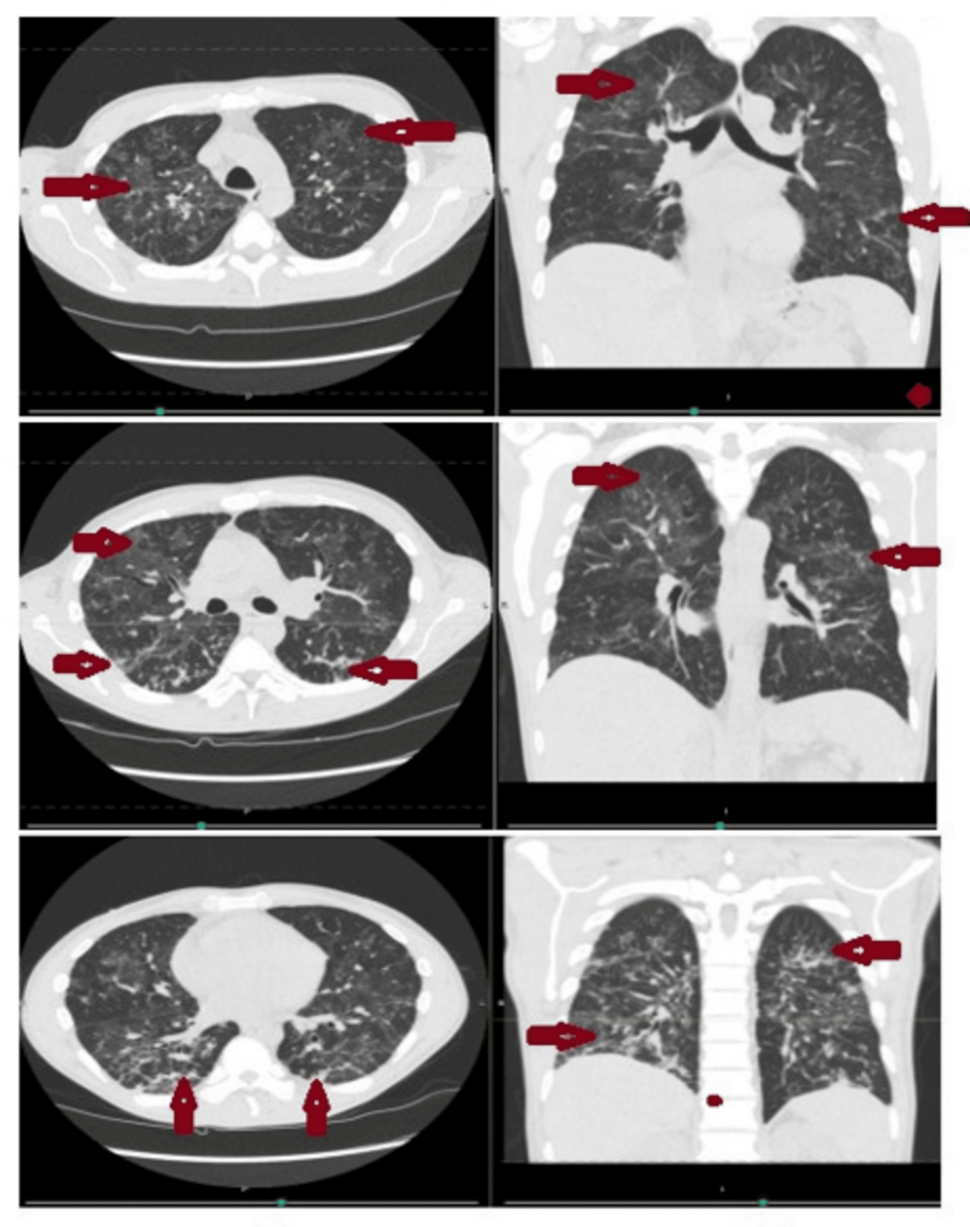
Axial and coronal views of chest CT scan without contrast at three different levels, showing improvement in bilateral consolidation, ground-glass, and nodular opacities, as indicated by the red arrows

**Figure 3:**
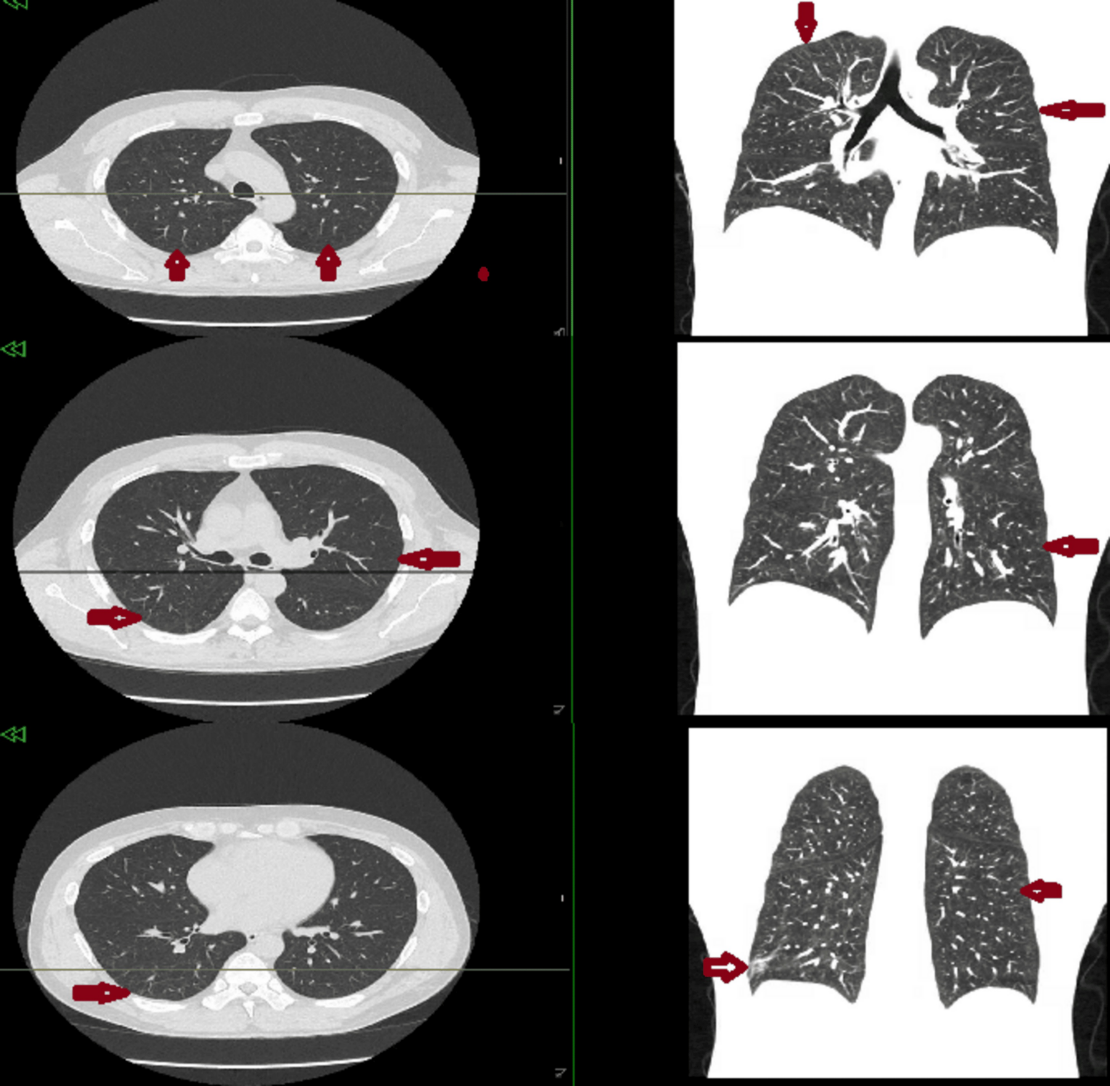
Axial and coronal views of chest CT scan without contrast at three different levels, highlighting interval resolution Red arrows mark the same previously involved areas for comparison.

A one-month follow-up chest CT showed marked improvement in the multifocal consolidations, ground-glass changes, and nodular opacities (Figure 2), with near-complete resolution demonstrated on the six-month follow-up CT scan (Figure 3).

## Discussion

NSIP is the most prevalent ILD pattern observed in MCTD and may occur in up to two-thirds of affected patients [2]. NSIP is characterized radiographically by ground-glass opacities, reticulations, and traction bronchiectasis, and histologically by temporally uniform inflammation with minimal architectural distortion [3]. These features differentiate NSIP from usual interstitial pneumonia (UIP), which is associated with worse outcomes and limited responsiveness to immunosuppression [4].

The development of ILD in MCTD is attributed to immune-mediated epithelial injury, chronic antigenic stimulation, microvascular dysfunction, and reflux-related microaspiration. Autoantibodies such as anti-U1 RNP, anti-Ro52, and anti-Sm are associated with a higher risk and greater severity of lung involvement [5,6]. Our patient’s serologic findings and family history align with previously described risk factors.

A major diagnostic challenge lies in distinguishing NSIP from infectious pneumonia. Both conditions may present with bilateral ground-glass opacities and respiratory failure; however, NSIP typically lacks significant leukocytosis, persistent fever, or microbiologic evidence of infection. The American Thoracic Society and European Respiratory Society (ERS) guidelines emphasize the importance of bronchoscopy with BAL to exclude infection before initiating high-dose steroids [7].

HRCT plays a pivotal role in NSIP diagnosis. Findings such as diffuse ground-glass opacities without honeycombing suggest an inflammatory phenotype that responds well to immunosuppression [3,8]. Furthermore, the updated ERS ILD nomenclature underscores the importance of identifying fibrosing versus inflammatory patterns early to guide long-term management [9].

Corticosteroids remain the first-line treatment for inflammatory NSIP. Steroid-sparing agents such as MMF or azathioprine are commonly used for long-term maintenance, supported by data demonstrating improved stabilization of lung function and reduced relapse [10,11]. Patients with rapidly progressive or refractory ILD may benefit from cyclophosphamide or rituximab [12]. Our patient’s rapid improvement following corticosteroids and mycophenolate is consistent with favorable outcomes reported for inflammatory NSIP in connective tissue disease-associated ILD (CTD-ILD) cohorts.

Prognosis varies by the extent of fibrosis and response to therapy. NSIP generally carries a more favorable prognosis than UIP, especially when treated early [2,13]. Ongoing surveillance with pulmonary function testing and HRCT is essential given the potential for relapse or fibrotic progression [14]. This case reinforces the importance of considering autoimmune ILD in patients with diffuse opacities and unexplained hypoxemia, particularly when they fail to respond to antibiotics [15].

## Conclusions

This case emphasizes the importance of considering CTD-ILD in patients presenting with diffuse ground-glass opacities, atypical respiratory symptoms, or failure to improve with appropriate antimicrobial therapy. NSIP is the most common ILD subtype in MCTD and may present acutely with severe hypoxemia. Early identification of autoimmune features, comprehensive serologic testing, and exclusion of infectious etiologies are essential steps in the diagnostic approach.

Prompt initiation of corticosteroids, along with appropriate steroid-sparing agents such as MMF or azathioprine, can lead to rapid clinical improvement, radiologic resolution, and prevention of irreversible fibrosis. Multidisciplinary collaboration among pulmonology, rheumatology, radiology, and pathology is crucial to optimize outcomes. Clinicians should maintain a high index of suspicion for CTD-ILD in cases of nonresolving pneumonia or unexplained hypoxemic respiratory failure, as early recognition and treatment can substantially alter disease trajectory and long-term prognosis.
